# Susceptibility of Dog, Hamster, and Mouse Cells to the Replication-Competent Adenovirus 11p E1/E3 Green Fluorescence Protein Vector Has Implications for the Selection of Animal Vaccine Models

**DOI:** 10.3389/fmicb.2021.698999

**Published:** 2021-10-27

**Authors:** Madhuri Gokumakulapalle, Li Wang, Ya-Fang Mei

**Affiliations:** Department of Clinical Microbiology and Virology, Umeå University, Umeå, Sweden

**Keywords:** RCAd11pGFP, E1/E3 vector, animal cells, GFP expression, cytotoxicity, DNA replication, susceptibility, QPCR assay

## Abstract

Human adenovirus (Ad)-vectored vaccines require viruses that can internalize into host cells and express the vaccine antigen. Evaluation of the expressed antigen in animal cells is a critical step in preclinical trials of viral vaccines. Due to the species specificity of Ads, it is difficult to find a suitable animal model. Thus, in this study, we compared the efficacy of Ad 11 prototype (Ad11p)-mediated green fluorescence protein (GFP) expression in cell lines of dog (MDCK), hamster (CHO), and mouse (McCoy and C127). Although these cell lines did not express the known primary cellular receptors for Ad11p virus infection (i.e., CD46), Ad11pE1GFP could infect and express GFP with various efficacies. For instance, it manifested relatively higher GFP expression in MDCK than in CHO, McCoy, and C127. However, infection leading to efficient viral release was not observed in any of the studied cell lines. The apparent differences were attributed to particularities of mouse and hamster cell lines, which might have led to the repression of viral DNA synthesis and to the low level of GFP expression mediated by Ad11pe3GFP. Moreover, our results revealed that undetectable hexon protein hampered the assembly of virus particles in CHO and MDCK cells. Ad11p differed from Ad5 in the ability for viral DNA synthesis when infecting CHO cells. Although a defective Ad has been successfully developed for SARS-CoV-2 vaccines in clinical applications, it has been difficult to generate one that can be used as an oral SARS-CoV-2 vaccine. Fortunately, our replication-competent Ad 11p vector might solve this problem. Regarding the use of Ad-vector candidates for vaccine purposes, this study demonstrates the selection of animal cell lines and determination of suitable virus doses in *in vitro* experiments.

## Introduction

As the world witnesses the coronavirus disease 2019 (COVID-19) pandemic caused by the severe acute respiratory syndrome coronavirus 2 (SARS-CoV-2), vaccines are urgently required to combat this disease. Currently, at least eight types of coronavirus vaccines, including inactivated whole virus particles or weakened virus vaccines, replicating and non-replicating virus-vectored vaccines, DNA or RNA vaccines, protein-subunit vaccines, and virus-like particle vaccines, have been designed, improved, and progressed to a certain extent (Callaway, 2020; Dong et al., 2020). Of these, the vaccines currently being widely used are the adenovirus (Ad)-vectored SARS-CoV-2 and mRNA vaccines (Folegatti et al., 2020; Logunov et al., 2020; Zhu et al., 2020).

Adenovirus vector-based vaccines have been developed using a strategy and possess unique properties that are distinct from those of classical protein vaccines, such as hepatitis B surface antigen (HBsAg) and inactivated polio vaccines, that can directly stimulate T cells and antibodies to fight against the virus (Horaud, 1993; Leroux-Roels et al., 1997). In contrast, the Ad of the vector-based vaccine enters the host cells and uses the machinery of those cells to express the viral antigen that it carries. This further stimulates the innate, humoral, and cell-mediated immune system of the host to fight viral infection. Thus, the detection of antigen expression in animal cells is important for evaluating the efficacy of the vaccine *in vitro*. Ads are host-specific, and human Ads can internalize into human cells and cause infection more efficiently than in cells from other species. Similarly, mouse and dog Ads manifest more effective infection in the cells of their original hosts than in human cells (Goffin et al., 2019). However, it is clear that at least certain cell lines from other species support the full replication cycle of Ads, despite showing reduced efficacy. Syrian hamsters and cotton rats are semi-permissive for Ad5 replication, which has made them useful animal models (Pacini et al., 1984; Thomas et al., 2006). Primary porcine cells have been shown to permit Ad5 replication (Jogler et al., 2006). Some murine epithelial cell lines can produce substantial amounts of infectious Ad2 virus, although at levels approximately 100-fold lower than those produced in human cells, which were included as positive controls. Interestingly, a report confirmed that a mouse mammary epithelial cell line supported highly efficient Ad2, Ad37, and Ad4 replication but not Ad12, Ad3, Ad11, and Ad41 replication (Wu et al., 2013). Thus, selection of susceptible animal cell lines is required for the evaluation of a new human Ad-vectored vaccine.

To date, the Ad vectors HAd5 and HAd26, or chimpanzee Ad expressing spike antigen, have entered clinical trials in China, Brazil, Russia, and the United Kingdom. These Ad vectors are non-replicating (defective), and the SARS-CoV-2 spike protein is inserted in the early region 1 (E1) of the viral genome. However, using defective viruses for vaccine purposes have disadvantages: (i) the viral genome is unstable and can be inactive after thermal treatment; (ii) these replication-defective vectors require a complementary cell line to produce defective virus particles (Bett et al., 1994; Kennedy and Parks, 2009; Smith et al., 2009), thereby taking longer and having a low yield. Electron microscopy revealed that defective virus particles seemed more unstable than variants with full-length genomic DNA (Mei et al., 2016). These results indicate the reduced efficacy of the currently used Ads that are replication-defective. Moreover, it has been difficult to generate a defective Ad for use as an oral SARS-CoV-2 vaccine, and there is evidence that the replication-competent vesicular stomatitis virus-vectored Ebola vaccine is more efficient than other Ebola vaccines (Halperin et al., 2019). Therefore, we believe that a replication-competent Ad expressing the COVID-19 spike protein could overcome the existing problems of developing an oral vaccine.

In our study, cell lines from different species were used to evaluate Ad11 prototype (Ad11p) vector-based transgene expression and then determine the susceptibility of animal cells to Ad11p infection. The green fluorescence protein (GFP) transgene was inserted in the E1 and E3 regions of the Ad11p genome. In these two cases, GFP expression was detected at various time points in cells from different animal species. To characterize the putative Ad vaccine, we compared the receptor and GFP expression levels, DNA replication, and viral replication in human, dog, hamster, and mouse cell lines infected with replication-competent Ad11pGFP vectors. Thus, this article describes the biological characteristics of the two expression vectors in four different host cells to aid in the selection of a suitable candidate animal model and viral dose for Ad11p-vectored vaccines in preclinical trials.

## Materials and Methods

### Cell Lines and Cell Culture

The A549 cell line from human oat-cell lung carcinoma was used in experiments to investigate viral DNA transfections, preparations, and infections. A549 cells were obtained from Dr. Waltel Nelson-Rees (Lieber et al., 1976) and cultivated as previously described (Wu and Mei, 2019).

Five cell lines were selected for this study: A498, derived from human renal carcinoma cells; MDCK, Madin and Darby canine kidney cells; CHO, Chinese hamster ovary cells; C127, murine mammary gland cells; and McCoy mouse fibroblast cells. These cell lines were grown in Dulbecco’s modified Eagle’s medium, except for McCoy cells that were cultured in Roswell Park Memorial Institute medium. The human cell line, A498, was supplemented with 10% fetal calf serum (FCS), while 5% FCS was added to the other cell lines. Additionally, the media for all cell lines were supplemented with 20 mM HEPES (pH 7.4), 0.75% NaCO_3_ (w/v), 100 U/ml penicillin, and 100 μg/ml streptomycin.

### Ad11p Vectors

Two replication-competent Ad11p vectors expressing the GFP gene in the E1 and E3 regions were generated according to Mei et al. (2016). These vectors were approved as European patents under registration number EP2486137B1 (registration date: 2018-05-30).

### Preparation of Virus Particles and Viral Genomic DNA

The infectivity and yield of the replication-competent Ad11p E1/E3 GFP vectors (RCAd11pe1GFP and RCAd11pe3GFP, respectively), and Ad11p and Ad5p viruses were investigated in A549 cells. In 175 cm^2^ flasks, 1 × 10^8^ A549 cells were infected with the RCAd11pe1, RCAd11pe3, or wild-type (Ad11pwt) virus over a course of 3–4 days; 8–10 flasks were used for each virus. To achieve complete cytopathic effect (CPE) of the viruses, the virus-infected cells were harvested by centrifugation (Eppendorf 5810R) at 800 rpm and 4°C for 5 min. Cells were then sonicated twice at 4°C for 10 s at 30% power. The cell lysate was extracted once with chloroform, and the viral supernatant was obtained after centrifugation of the lysate at 4,000 rpm and 4°C for 1 min. Subsequently, gradient ultracentrifugation was performed as previously described (Mei et al., 1998, 2016; Havenga et al., 2002). As shown in Table 1, the viral yield from the RCAd11pe1GFP and RCAd11pe3GFP cultures was compared to that of Ad11pwt. The infectivity of the viral particles was determined by measuring the 50% tissue culture infection dose (TCID_50_) in A549 cells as previously described (Wu and Mei, 2019). Viral genomic DNA was prepared from infected cells or purified virions, according to previously described methods (Shinagawa et al., 1983).

**TABLE 1:** Characteristics of replication-competent adenovirus 11p E1/E3 GFP as expression vectors in dog, hamster, and mouse cells.

**Cell lines**	**CD46 Mono-Ab**	**CD46 Poly-Ab**	**avb3**	**avb5**	**E1GFP vector**	**E3GFP vector**	**DNA synthesis**	**Cytotoxicity assay**	**Progenic viruses**
A498	+++	+++	+++	+++	++++	++++	Yes	Yes	Yes
MDCK	–	–	–	–	++++	++++	Yes	No	No
CHO	–	–	–	+	++++	+	No	No	No
McCoy	–	–	–	–	+	±	No	No	No
C127	–	–	–	–	+	–	No	No	No

*++++, most strong; +++, very strong; ++, strong, +, clear positive; ±, weak positive; no, undetectable.*

### Analysis of the Receptor Molecules on the Surface of Human and Animal Cells

Overnight cultivated cells from each cell line were washed once with phosphate-buffered saline (PBS) containing EDTA and then detached using 0.1% trypsin in PBS/EDTA. The cell pellets were allowed to recover at 37°C for 1 h with slow shaking in culture medium containing 2% FBS. Thereafter, the cells were counted and centrifuged at 1,500 rpm at 4°C for 5 min. The cell pellets were then suspended in 100 μl binding buffer (PBS with 2% FBS and 0.01% NaN_3_) at a concentration of 2 × 10^5^ cells/100 μl and transferred into a 96-well microtiter plate, which was then centrifuged at 800 rpm and 4°C for 5 min and kept on ice. The expression of the cell surface molecules CD46, ανβ3 integrin, and αvβ5 integrin, was detected using the following antibodies: mouse monoclonal anti-human CD46 (169-1-E4.3, Ancell, Bayport, MN, United States) diluted 1:200, rabbit polyclonal anti-CD46 (H-294; Santa Cruz Biotechnology Inc., Santa Cruz, CA, United States) diluted 1:200, anti-human integrin αvβ3 (MAB1976; Chemicon, Temecula, CA) diluted 1:200, and anti-human integrin αvβ5 (MAB1961; Chemicon) diluted 1:200. The pellets were resuspended in 100 μl of monoclonal antibodies diluted in binding buffer, and the mixtures were incubated for 30 min on ice. The cell pellets were washed again with binding buffer and resuspended in 100 μl of fluorescein isothiocyanate (FITC)-conjugated goat anti-mouse Fab (Sigma, St. Luis, MO, United States) or FITC-conjugated swine anti-rabbit antibody (F0205; DAKO, Carpinteria, CA, United States), which was diluted 1:100 in binding buffer. The cells were then incubated on ice in the dark for 30 min. The pellets were washed again, resuspended in 300 μl PBS, and finally transferred to Falcon tubes for fluorescence-activated cell sorting (FACS) analysis.

### Ad11p Receptor Blocking Assay

To determine whether cells from different origins express the viral receptor for Ad11p infection, recombinant Ad11p fiber knob (Ad11prfib) was used to block the cell receptor (Mei and Wadell, 1996). Cells were plated at a density of 2 × 10^5^ cells/well in a 24-well plate, and Ad11prfib was added at 0.1, 1.0, or 10 pg/cell. The cells were then incubated at 4°C for 1 h. The RCAd11pe1GFP vector was added to the cells at concentrations of 0.1, 1.0, or 10 pg/cell, and the cells were incubated at 37°C for an additional hour. The cells were then washed twice with 2% bovine serum albumin (BSA) in PBS, and fresh culture medium was added. GFP fluorescence was measured by flow cytometry at 48 h post infection (h.p.i.).

### Fluorescence Microscopy and Fluorescence-Activated Cell Sorting Analysis

Cells were seeded at a density of 2 × 10^5^ cells/well in a 24-well plate and incubated at 37°C overnight. The cells were infected with RCAd11pe1GFP or RCAd11pe3GFP viruses at 0.1, 1.0, or 10 pg/cell for 1 h. GFP expression in each cell line was monitored daily using fluorescence microscopy.

The transduction efficiencies of the RCAd11pe1GFP and RCAd11pe3GFP viruses in the animal cell lines were compared using FACS analysis. Cells were seeded at a density of 2 × 10^5^ cells/well in 24-well plates. The next day, the semi-confluent cells were transduced with the RCAd11pe1GFP or RCAd11pe3GFP viruses at 0.1, 1.0, or 10 pg/cell for 1 h; mock-infected cells served as negative controls. The cells in the 24-well plates were washed once with culture medium and then incubated with the same medium for 48 h at 37°C. The plates were then centrifuged at 800 rpm at 4°C for 5 min. The cells were then fixed in 0.1 ml 4% paraformaldehyde (PFA) for 10 min at room temperature (20°C) and washed once with PBS containing 2% FBS. Thereafter, the cells were resuspended in 300 μl of the same buffer, and GFP expression was analyzed using a FACScan cytometer (Becton Dickinson Bioscience, Heidelberg, Germany). A total of 10,000 events were collected for each experiment. To distinguish dead cells from infected and uninfected cells, propidium iodide (PI) solution was added to the FACS tubes. The percentage of GFP-expressing cells was measured using flow cytometry.

### Quantitative Real-Time PCR Assay for the *hexon* Gene

Virus replication in human and animal cell lines was also detected by an established quantitative real-time PCR (qPCR) method (Andersson et al., 2010) in our virus lab. Virus infection in animal cell lines was carried out as described in our recent publication (Gokumakulapalle and Mei, 2016).

### Ad11p and Ad5 Viral DNA Preparation

To further study the differences in Ad5 and Ad11p abortive infection in animal cells, Ad11pwt, Ad5, and pFG140 were used to infect animal and human A498 cells. pFG140 is a replication-competent Ad5 vector with E1 partial deletion (Bett et al., 1994). The cells were infected with 1 pg/cell and cultivated for 2, 4, or 6 days at 37°C. Viral DNA was extracted as described previously (Shinagawa et al., 1983) and digested with the *Bam*HI restriction enzyme. Viral DNA bands were separated on a 1% agarose gel at 50 V overnight.

### Toxicity Assay

Cells from each cell line were seeded at a density of 2 × 10^5^ cells/well in 24-well plates and grown until they reached 85% confluence. The cells were then infected with Ad11pwt, RCAd11pe1GFP, or RCAd11pe3GFP at 0.001, 0.01, 0.1, or 1.0 pg/cell. The plates were stained every second day until day 12 p.i. The cell medium was removed, and the cells were fixed with 4% paraformaldehyde for 10 min at room temperature (20°C). The cells were then washed with PBS and stained with 1% crystal violet and 70% ethanol for 10 min. After staining, the cells were rinsed three times with water and air-dried before photographs were captured.

### Immunodetection of Hexon Protein Expression

Infections were carried out according to the method described by Wu and Mei (2019). The expression of hexon protein in animal cell lines mediated by RCAd11pE1GFP and RCad11pE3GFP was quantified using the Trophos assay. At 24, 48, and 72 h.p.i., the number of positive cells was detected by Ad11p virion antibody (KS590, virus lab at UmU) at 1:1,000 dilution in PBS; cells were shaken at 4°C for 1 h and then washed three times with PBS. The cells were visualized using polyclonal goat anti-rabbit IgG (H + L) secondary antibody, Alexa Fluor 647 (1:500 dilution in PBS), and DAPI (1:5,000 dilution) for 30 min at 4°C. The cells were then washed three times with PBS, and fluorescence was measured using a Trophos plate reader.

### Statistical Analyses

The *t*-test and one-way ANOVA was performed to test differences among cell lines and vectors on GraphPad Prism software (version 7.0; Graph Pad Software, San Diego, CA, United States). *p* < 0.05 was considered significant.

## Results

### Dog, Hamster, and Mouse Cells Did Not Express the Ad11p Primary Receptor CD46

**FIGURE 1:**
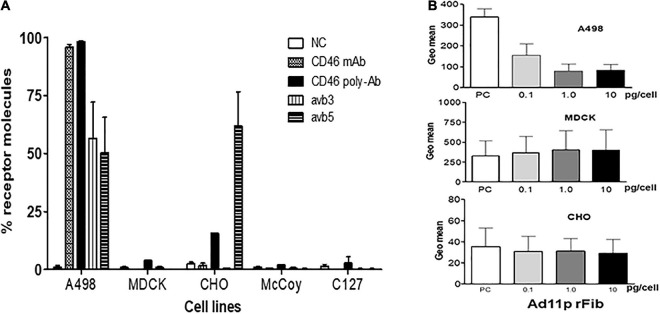
Receptor molecules on human and animal cells for infection with Ad11p virus detected using FACS analysis on human and animal cells. **(A)** Expression of the cell surface markers, CD46, and integrins ανβ3 and ανβ5, determined in the studied cell lines. The cells were incubated with antibodies against monoclonal or polyclonal CD46, integrin ανβ3, and integrin ανβ5. The expression of the relevant cell surface molecules on the animal cells studied was determined by flow cytometry analysis. The experiments were performed in duplicate, and at least three independent experiments were performed. **(B)** The Ad11p-fibre receptor on the MDCK and CHO cells was further inhibited by the addition of the Ad11prfib protein; A498 cells served as the positive control. A dose-dependent reduction of GFP expression in response to Ad11prfib protein was observed in A498 cells but not in MDCK and CHO cells.

To clarify whether animal cells expressed the receptor molecules responsible for Ad11p binding and uptake, the levels of human CD46 and the expression of integrins, ανβ3, and ανβ5, were monitored. The percentage of CD46 molecules detected using monoclonal anti-CD46 antibody differed remarkably on the cell surfaces of the four cell lines; CD46 was detected in 98% of A498 cells but was undetectable in MDCK, CHO, McCoy, and C127 cells (Figure 1A). However, when the polyclonal anti-CD46 antibody was employed, the CD46 expression level was low (15%) in CHO cells and barely detectable in MDCK and C127 cells (3 and 1%, respectively) (Figure 1A). These findings indicated that the animal cells employed in this study were deficient in CD46, despite a slight cross-reaction with the polyclonal anti-CD46 antibody in CHO cells. Integrins αvβ3 and αvβ5 were not markedly expressed above the detection limit in the animal cells, except in CHO cells that expressed a relatively high level of integrin αvβ5 (60%), but there was no detectable integrin αvβ3. Thus, the results revealed that human A498 cells, but not animal cells, express CD46 cell surface receptors that enable RCAd11p infection.

### No Detectable Fiber Receptor for Ad11p Infection in Dog, Hamster, and Mouse Cells

To ascertain whether Ad11p fiber receptors present on human cells existed on the surface of animal cells, we used Ad11pfib to block RCAd11p infection in A498, MDCK, and CHO cells, and the frequencies of the infected cells were detected using FACS analysis. When increasing concentrations of Ad11pfib were added, GFP expression was reduced in A498 cells, but not in MDCK and CHO cells. These results supported the hypothesis that the MDCK and CHO cell lines lacked the primary fiber receptor for Ad11p infection (Figure 1B).

### RCAd11pe1GFP and RCAd11pe3GFP Were Efficiently Produced in A549 Cells

**FIGURE 2:**
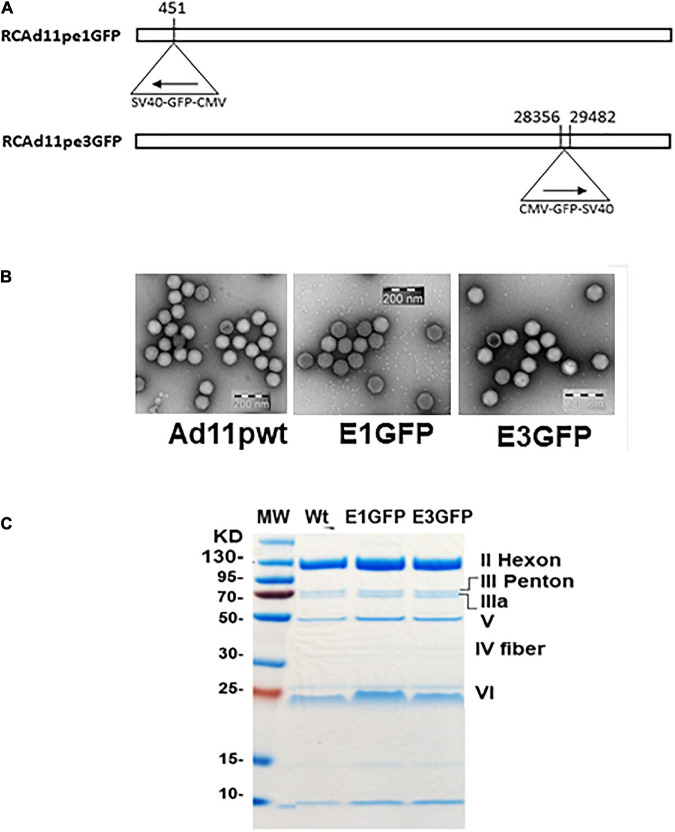
**(A)** Strategy for the development of replication-competent Ad11p containing the GFP gene in early regions 1 and 3 (RCAd11pe1GFP and RCAd11pe3GFP). **(B)** Electron microscopy images of virus particles. **(C)** Viral protein analysis using SDS-PAGE and Coomassie brilliant blue staining.

Both Ad11p vectors were propagated in A549 cells and purified by CsCl ultracentrifugation (Gokumakulapalle and Mei, 2016; Mei et al., 2016). Thereafter, the viral titers were evaluated, and the morphologies were determined using electron microscopy and SDS-PAGE (Figures 2A–C). The ratio of infectious to total virus particles (IP/VP) was expected to reach ≥1:72 to proceed with the use of the virus in subsequent validation experiments.

### Green Fluorescence Protein Expression at the E3 Region Was Dependent on Viral DNA Replication

**FIGURE 3:**
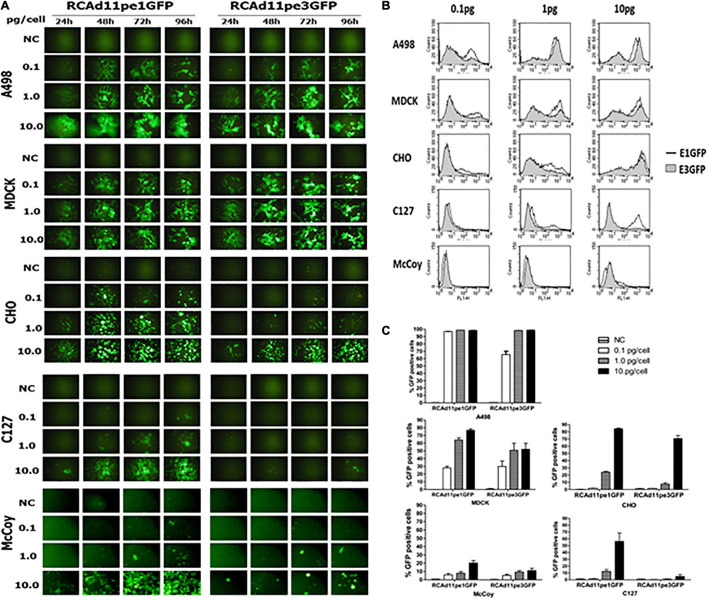
The abilities of the RCAd11pe1GFP and RCAd11pe3GFP viruses to infect human, dog, hamster, and mouse cells are compared. **(A)** Cells were infected with various amounts of RCAd11pe1 or RCAd11pe3 vectors and incubated for 24, 48, 72, or 96 h. GFP expression was assayed daily in each cell line using fluorescence microscopy. Images obtained using fluorescence microscopy at 200 × magnification. **(B)** The percentage of GFP-expressing cells with 0.1, 1, and 10 pg virus/cell was assessed using flow cytometry at 48 h.p.i. Results are from at least three independent experiments. **(C)** Comparison of GFP expression mediated by RCAd11pe1 or RCAd11pe3 vector, as shown by the histogram overlay. Open profile indicates RCAd11pe1GFP whereas the shaded profile represents RCAd11pe3GFP.

A GFP expression cassette controlled by the cytomegalovirus promoter was inserted into the E1 or E3 region of the Ad11p genome. The fluorescence intensity mediated by RCAd11pe1 or RCAd11pe3 GFP was compared and quantified using flow cytometry in both human and animal cells. GFP expression by both vectors was similar for A498 and MDCK cells, and it was detectable even at low doses of viral particles (0.1 pg/cell). GFP was highly expressed in both cell lines at 1 or 10 pg/cell (Figures 3A–C). However, CHO cells infected with the RCAd11pE3 vector exhibited 10-fold lower GFP expression than those infected with the RCAd11pE1 vector. In contrast, after infection with the same dose of virus in the two mouse cells, GFP expression from the E3 vector was 100-fold lower than that from the E1 vector. Thus, it was evident that the location of the GFP cassette upstream or downstream of the Ad11p genome determined transduction efficiency.

### Fold Increases of Viral DNA Copies in MDCK and Human A498 Cells

**FIGURE 4:**
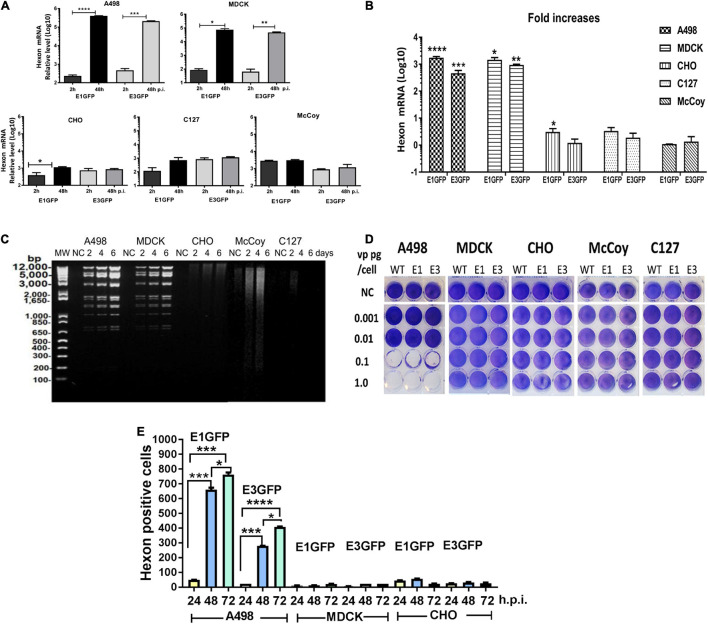
Detection of virus replication in animal cell lines. **(A)** Quantification of Ad11p *hexon* gene expression in animal and human cells was measured to quantify progeny DNA at 48 h.p.i. **(B)** Fold increases in DNA copies. The asterisks indicate a significant difference, as determined by an unpaired *t*-test, *p < 0.05; **p < 0.01; ***p < 0.001. The data are means ± SEs from three samples. **(C)** Detection of Ad-specific restriction enzyme fragments in the DNA from RCAd11pe1GFP-infected animal cells at different time points. Total viral DNA was extracted from one 175-cm^2^ flask for each infected cell line at 48, 96, or 144 h. Purified viral DNA was digested with *Bam*HI; digested lambda DNA was used as a molecular weight (MW) marker; the corresponding lengths are indicated. **(D)** Toxicity assays were performed to determine the productive infection of the viruses. RCAd11pe1GFP resulted in different cytolytic effects in human and animal cells. **(E)** Quantitative hexon expression in A498, MDCK, and CHO cells at 24, 48, and 72 h.p.i. The number of hexon-positive cells was quantified by using a Trophos plate reader. The asterisks indicate a significant difference, as determined by an unpaired *t*-test, *p < 0.05; **p < 0.01; ***p < 0.001. The data are means ± SEs from three samples.

The replication capacity of Ad11pGFP vectors in animal cells was detected by qPCR assay according to Andersson et al. (2010). We quantified *hexon* gene expression at 48 vs. 2 h.p.i., as Ad replication usually occurs after 2 h in infected cells. In human A498 and MDCK cells, approximately 3 logs of hexon mRNA copies were significantly increased for both RCAd11pe1 and RCAd11pE3GFP vectors. In contrast, we found no significant increase in Ad11p hexon mRNA copies in CHO, MDCK, and C127 cells, with mRNA copies appearing similar to the starting time point (Figures 4A,B).

### Full-Length of Ad11p Viral DNA Was Synthesized in MDCK Cells Only

To elucidate why GFP expression varied remarkably among the different cell types, we further investigated the DNA restriction enzyme pattern. Viral DNA isolated from the five cell lines was digested with *Bam*HI and stained with ethidium bromide; viral DNA bands were observed in A498 and MDCK cells at 2 days p.i., which further increased in intensity. In contrast to MDCK and A498 cells, no DNA was synthesized at any of the indicated time points in CHO, McCoy, and C127 cells (Figure 4C). These results indicated that the viral DNA was efficiently synthesized in A498 and MDCK cells but not in CHO cells, where a smeared DNA pattern was observed; viral DNA was only marginally produced in McCoy and C127 cells (Figure 4C).

### No Cytolytic Effect of RCAd11pGFP on Dog, Hamster, and Mouse Cells

To determine which cell types could produce progeny viral particles, Ad11pwt, RCAd11pe1, and RCAd11pe3 viruses at concentrations of 0.001, 0.01, 0.1, or 1.0 pg/cell were used to infect the five cell lines, and toxicity assays were performed. At 12 days p.i., the cytolytic effects on the cells were visualized by staining with crystal violet. Ad11pwt and RCAd11p vectors efficiently induced cytolytic effects in A498 cells at 0.1 pg/cell. In contrast, 1 pg/cell of viruses had a limited effect on MDCK cells and had no effect on CHO, McCoy, and C127 cells (Figure 4D).

### Undetectable Hexon Expression in CHO and MDCK Cells

The expression of the structural hexon protein was further investigated in the three cell lines infected with the two Ad11p GFP vectors. Hexon protein was highly expressed in human A498 cells but not in CHO and MDCK cells. In general, the number of hexon-positive cells mediated by E1GFP was higher than that by the E3GFP vector at 24, 48, and 72 h.p.i. (Figure 4E). Therefore, our results revealed that the viral hexon protein hampered the assembly of virus particles in CHO and MDCK cells (Table 1).

### CHO Cells Can Synthesize the Ad5 Genome but Not the Ad11pwt Genome

**FIGURE 5:**
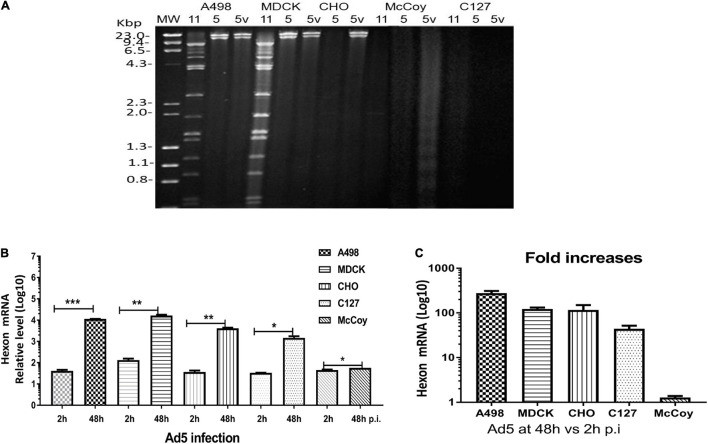
**(A)** Assessment of Ad11p, Ad5p, and pFG140 viral DNA replication in A498, MDCK, CHO, McCoy, and C127 cell lines. Animal cells (MDCK, CHO, McCoy, and C127) and the control human (A498) cells were infected with Ad11p, Ad5, and pFG140 at 1 pg/cell, as described in section “Materials and Methods.” Viral DNA harvested and purified from the infected cells was digested with the *Bam*HI restriction endonuclease. Sample list: **11** means Ad11p; **5** means Ad5p; **5v** means pFG140 vector. **(B)** Quantification of Ad5 hexon expression at 48 vs. 2 h.p.i. **(C)** Fold increases in DNA copies in four animal cell lines and human A549 cells. The asterisks indicate a significant difference, as determined by an unpaired *t*-test, *p < 0.05; **p < 0.01; ***p < 0.001. The data are shown as averages ± SEs from three samples.

To understand the differences between the cells infected with Ad11p and Ad5, we further investigated the replication properties of Ad11p, Ad5p, and Ad5 vectors (pFG140) in the animal cells compared to the human A498 cells. The cell lines were infected with virus at 1 pg/cell, and after 4–6 days, the viral DNA was extracted and digested with the *Bam*HI restriction enzyme. The restriction patterns of Ad11p and Ad5p DNA isolated from A498 and MDCK cells were similar; however, those isolated from infected CHO cells were significantly different. CHO cells produced Ad5p, but not Ad11p viral DNA. No viral DNA was isolated from Ad11p- or Ad5p/pFG140-infected McCoy and C127 cells; only faint and degraded smears were observed. These results revealed that the replication of viral DNA from Ad11p, Ad5p, and pFG140 viruses in MDCK cells was similar to that in A498 cells during the observation period (Figure 5A). However, Ad11p did not synthesize a full genome in CHO cells, but Ad5p did. Even though the mouse CAR receptor is expressed in mouse cells (Roelvink et al., 1998), McCoy and C127 cells, which are of mouse origin, were clearly not susceptible to Ad11p, Ad5, and pFG140 infection in the restriction pattern (Figure 5A). Nevertheless, the qPCR assay allowed detecting Ad5 hexon mRNA copies in C127 and McCoy cells at 48 h.p.i. (Figures 5B,C).

## Discussion

Adenovirus vectors have been safely used in several vaccines against emergent diseases, including influenza, malaria, Ebola, and HIV. Ad-vectored vaccines are a modern technology that can be quicker than classical inactive/attenuated virus vaccines or protein subunit vaccines. However, despite progress, more efficient Ad-vectored vaccines should be developed for clinical application. One of the critical factors is that Ad has host specificity—human Ads are less efficient at infecting animal cells. Thus, a new human Ad serotype as a vectored vaccine requires the selection of suitable animal cells and effective virus doses to carry out antigen expression *in vitro*.

Cells can be susceptible, semi-susceptible, or not susceptible to Ad infections. Therefore, we initiated the screening of suitable cell lines in which Ad could be internalized, resulting in productive or abortive infection. MDCK cells are commercially used for the propagation of human influenza virus (Katz and Webster, 1989); however, this cell line has not been used for the propagation of human Ad. In our study, although MDCK cells did not express surface molecules, such as CD46 or integrins ανβ3 and ανβ5, RCAd11pGFP vectors could infect the cells as efficiently as they infected the human A498 cells. These results indicate that other uncharacterized molecules, but not CD46, act as the primary receptor for Ad11p in MDCK cells. RCAd11pGFP infected CHO, C127, and McCoy cells via a CD46-independent pathway, as detected by both polyclonal and monoclonal anti-CD46 antibodies. No human CD46 analogs or integrins were detected in the animal cells, except in CHO cells. The fiber-blocking assay showed that there was no detectable fiber receptor for Ad11p infection, suggesting that the lack of a receptor was not a major obstacle for viral infection, and the infection was most likely mediated by alternative receptor analogs.

The amount of viral DNA template directly affected GFP expression in infected cells. Low amounts of viral DNA copies showed low levels of GFP expression, as indicated by the two vectors carrying identical expression cassettes located either upstream of the E1 region or downstream of the E3 region of the RCAd11p genome. After infection with the two vectors, GFP expression in MDCK cells was similar to that in A498 cells. The viral DNA and toxicity assays indicated that the abortive Ad11p infection in MDCK cells occurred at a later stage, presumably during virus packaging or release. In contrast, CHO, McCoy, and C127 cells expressed significantly lower GFP (10–100-fold lower than the levels observed in MDCK or A498 cells) when infected with RCAd11pe3 than when infected with RCAd11pe1, which was dependent on viral DNA replication (Figure 4A). In addition, a high dose of RCAd11pGFP (10 pg/cell) only resulted in infection in 20% of the cells expressing GFP. This supports the notion that mouse cells are less susceptible to RCAd11pGFP infection.

The replication-competent Ad11p vector with the insertion of a marker gene at the 5’ end of the Ad11p genome (E1 region) did not disrupt viral transcription or replication (Mei et al., 2016). These findings are especially important for the use of viruses as expression vectors for vaccine purposes because conventional defective vectors with the deletion of the E1 genes followed by the insertion of a transgene results in considerably reduced transactivation, transcription, and virus stability (Kennedy and Parks, 2009). Consequently, the replication-competent Ad11p vector can be used efficiently and precisely to investigate the susceptibility of animal cells to the virus of interest while the defective vector cannot.

Our findings demonstrated that Ad11 caused early abortive infection in McCoy and C127 cells. Moreover, we found that Ad11p DNA synthesis was blocked in CHO cells but that of Ad5 DNA was not. It is possible that the DNA polymerases differed between these two viruses. Our findings are in agreement with those of an earlier study in which CHO cells produced amounts of Ad2 DNA that were similar to the levels synthesized in permissively infected human cells; however, the viral structure protein was undetectable (Longiaru and Horwitz, 1981).

We demonstrated that CHO and mouse cells did not support Ad11p genome replication, whereas CHO cells supported Ad5 genome replication. In MDCK cells, RCAd11p efficiently replicated and produced genomic DNA; however, there was very limited or undetectable expression of the structural hexon protein, resulting in the failed assembly of other viral structure proteins (Figure 4E). The typical characteristics of the RCAd11p vector with a foreign gene expressed in the E1 region of the Ad11p genome could be beneficial for vaccine purposes, as viral DNA and structural proteins are blocked and only the inserted vaccine gene can be efficiently expressed in animal cells, resulting in limited anti-Ad11p hexon antibody being induced *in vivo*. Therefore, the RCAd11p vector has remarkable advantages for the assessment of candidate immunogens in animal cell lines.

The replication of human Ads is restricted to human cells. However, it is evident that at least certain cell lines from other species support the full replication cycle of Ads, despite their reduced efficacy. Syrian hamsters and cotton rats are semi-permissive for Ad5 replication, which has made them useful animal models (Thomas et al., 2006). Primary porcine cells have been shown to permit Ad5 replication (Jogler et al., 2006). Some murine epithelial cell lines can produce substantial amounts of infectious Ad2 virus, although at levels approximately 100-fold lower than those produced in the human cells included as positive controls. Interestingly, a report confirmed that a mouse mammary epithelial cell line supported highly efficient Ad2, Ad37, and Ad4 replication but not Ad12, Ad3, Ad11, and Ad41 replication (Wu et al., 2013).

In this study, we described the characteristics, including GFP expression and viral genome DNA replication, of replication-competent Ad11p vectors infecting animal cells. Results implied that replication-competent Ad vectors may be used in animal experiments for vaccine purposes. Moreover, the replication-competent Ad11p vector with E1 insertion has more advantages than that with E3 insertion. Dog, hamster, and mouse cells could be selected for use in antigen expression tests involving Ad11p-vectored vaccines; however, a relatively high dose of the Ad11p vector is suggested in mouse models because of the host specificity issue. Replication-competent human Ad4 and Ad7 have been used as oral vaccines to protect US soldiers against severe respiratory diseases caused by these viruses since the 1970s. These vaccines are thought to establish a digestive tract infection that confers protection against respiratory challenge through antibodies. The success of these vaccines makes replication-competent Ads attractive candidates for use as oral vaccine vectors (Gurwith et al., 2013; Hoke and Snyder, 2013; Matsuda et al., 2021). A defective AdV has been successfully developed for SARS-CoV-2 vaccines in clinical applications. However, a defective Ad for use as an oral SARS-CoV-2 vaccine has not been identified so far. The ultrastructure of defective AdV is poor and real functional virus particles are limited. A defective vector usually needs 10 times more virus particles to internalize the host cells than its parent virus particles. In addition, viruses should be resistant to Trypsin and chymotrypsin in order to reach to the intestine epithelial cells for the antigen expression. Furthermore, the yield for defective AdV vaccine requests is very high, which would be difficult to generate. Furthermore, in comparison with replication-defective vectors, replication-competent adenovirus vectors may have an enhanced ability to break immune tolerance mechanisms present in the gut mucosa (Deal et al., 2013). Results of this study indicate that the replication-competent Ad11p vector may solve the problems of the defective vector. Thus, our research lays the foundation for the use of the Ad11p-vectored vaccine in animal cells *in vitro* before preclinical trials.

## Data Availability Statement

The original contributions presented in the study are included in the article/supplementary material, further inquiries can be directed to the corresponding author.

## Author Contributions

MG, LW, and Y-FM performed the experiments and interpreted the data. Y-FM designed and performed the study and wrote the manuscript. All authors contributed to the article and approved the submitted version.

## Conflict of Interest

The authors declare that the research was conducted in the absence of any commercial or financial relationships that could be construed as a potential conflict of interest.

## Publisher’s Note

All claims expressed in this article are solely those of the authors and do not necessarily represent those of their affiliated organizations, or those of the publisher, the editors and the reviewers. Any product that may be evaluated in this article, or claim that may be made by its manufacturer, is not guaranteed or endorsed by the publisher.

## Abbreviations

Ad11p: Human adenovirus 11 prototype
Ad5p: Human adenovirus 5 prototype
AdV: Adenovirus vector
RCAd11p: replication-competent adenovirus 11p
GFP: green fluorescence protein
E1GFP: GFP expression cassette at early region 1 of Ad11p genome
E3GFP: GFP expression cassette at early region 3 of Ad11p genome.
